# Clinico-Epidemiological Profile of Dengue in Children of Age Group 0-14 Years Admitted in a Tertiary Care Hospital

**DOI:** 10.7759/cureus.87027

**Published:** 2025-06-30

**Authors:** Sukriti Mishra, Ravi Ambey, Ayush Khare, Ajay Gaur

**Affiliations:** 1 Paediatrics, Gajra Raja Medical College, Gwalior, IND

**Keywords:** bleeding manifestation, dengue in children, dengue without warning signs, dengue with warning signs, non-severe dengue, pediatric dengue, severe dengue, thrombocytopenia, tourniquet test

## Abstract

Background

Dengue has emerged as the most common arboviral infection in India and worldwide. Dengue presents with a varied clinical spectrum in different age groups. The changing epidemiology and presentation are a challenge for early diagnosis and management. This prospective observational study aimed to determine the epidemiological profile of hospitalized dengue-positive children aged 0-14 years and correlate their clinical and hematological profiles.

Methodology

One hundred and five children with laboratory-confirmed dengue were enrolled in this study over a six-month study period. They were categorized into severe and non-severe dengue according to the World Health Organization (WHO) classification. Detailed clinical examination and laboratory investigations, including complete blood count, liver enzymes, coagulation profile, and imaging (chest X-ray and abdominal ultrasound), were performed. Data were analyzed using descriptive statistics, chi-square test, and logistic regression analysis.

Results

Out of the total 105 cases, 90 children (85.7%) had non-severe dengue and only 15 (14.3%) had severe dengue. The mean age of children was 92.02 months, with a male predominance (72, 68.6%). The most commonly identified symptoms were fever (105, 100%), vomiting (47, 44.76%), and abdominal pain (46, 43.81%). It was found that although thrombocytopenia was seen in 79 children (75.2%), it was not significantly associated with the severity of the disease (*P* = 0.2364). The study found a high recovery rate (101, 96.3%) with a mortality of 3 patients (2.8%) in severe cases. Elevated serum glutamate pyruvate transaminase (SGPT) levels (*P* = 0.0025), pleural effusion on chest X-ray (*P* = 0.000452), abdominal pain (*P* = 0.0041), and a positive tourniquet test (*P* < 0.0001) were found to be significantly associated with severe dengue. Non-structural protein 1 (NS1) antigen and immunoglobulin M (IgM) antibodies were both found to be positive more frequently in severe cases compared to non-severe cases (*P* = 0.0361).

Conclusions

This study observed a high rate of recovery and low mortality, owing to the timely management. A possible shift in the seasonal distribution of dengue was observed, with many cases occurring in the winter season. While thrombocytopenia was prevalent, it was not found to be significantly associated with disease severity or bleeding. On the other hand, elevated liver enzymes, pleural effusion, abdominal pain, and a positive tourniquet test were found to be more strongly associated with severe dengue. They may serve as valuable early clinical indicators for the severity of the disease.

## Introduction

Dengue is a vector-borne viral infection with rising trends seen in its incidence worldwide. It is caused by the dengue virus (DENV), which comprises four distinct serotypes (DENV-1 to DENV-4) and is transmitted by Aedes mosquitoes, which are well-adapted to urban environments and increasingly rural settings [1].

In 2024, more than 14 million dengue cases and over 10,000 dengue-related deaths were reported globally [2]. Dengue in India has expanded largely over the last few decades, with rapidly changing epidemiology. A possible shift in the seasonal distribution of dengue was observed, with many cases occurring in the winter season. These changing trends may be attributed to a combination of epidemiological factors - prolonged monsoon and post-monsoon humidity, rising temperature, increased population density, urbanization with poor drainage, and water accumulation. Dengue is known to be endemic in most of the states of India [3]. This rise in dengue cases may be attributed to various factors like urbanization, climate change, population drift, genetic mutations in the virus, and the absence of an effective vaccine [4].

Clinical profile of dengue ranges from a self-limiting illness to life-threatening dengue hemorrhagic fever (DHF) or dengue shock syndrome (DSS), which have mortality rates as high as 20% [5]. In recent years, India has witnessed changing trends in pediatric dengue. With rising disease burden, atypical manifestations like neurological involvement, hepatic dysfunction, acute kidney injury, myocarditis, and other isolated organ involvement, constituting the expanded dengue syndrome, are also on the rise, but are underreported and often missed [6]. Several poor prognostic markers have been identified, which indicate progression to severe forms and high mortality [7]. These markers are particularly important in pediatric patients, in whom disease progression can be rapid and less predictable. Early diagnosis of dengue allows timely management and can save lives. Despite advances in diagnostics, early recognition of severe dengue remains a challenge in pediatric practice, particularly in resource-limited settings.

Witnessing the changing trends in the clinical profile of dengue in children, this study aims to assess the changing epidemiology and clinical spectrum of pediatric dengue in central India, with the following objectives:

(1) To determine the epidemiological profile of hospitalized dengue-positive children aged 0-14 years

(2) To correlate the clinical and hematological profiles of hospitalized dengue-positive children aged 0-14 years

## Materials and methods

Study design

This prospective, observational, facility-based study was conducted at a tertiary care hospital of a government medical college in Central India. The objective was to determine the epidemiological profile of hospitalized dengue-positive children aged 0-14 years and to correlate their clinical and hematological profiles.

Study population and sample size

A total of 105 hospitalized dengue-positive children aged 0-14 years were included in the study.

Inclusion and exclusion criteria

Children aged 0 to 14 years who were admitted to a tertiary care hospital during the study period (July 2024 to December 2024) with a confirmed diagnosis of dengue infection (positive for IgM, NS1, or both) and whose parents provided informed consent were included in the study. Children whose parents did not provide consent for participation in the study were excluded.

Ethical approval

Ethical approval for the study was obtained from the Institutional Ethical Committee of the institute (Reference No. 1373/IEC-GRMC/2024, May 1, 2024).

Methodology

No identifying information, such as name and address, was collected. Upon admission, baseline data, including demographic details, medical history, findings from a thorough clinical examination, and results of baseline investigations, were recorded. Cases were classified as per the World Health Organization (WHO) 2009 classification into dengue without warning signs, dengue with warning signs, and severe dengue. Throughout the hospital stay, patients were closely monitored through regular clinical assessments, vital sign tracking, and charted on a monitoring sheet. Daily hematocrit, platelet count, liver function test, prothrombin time, international normalized ratio (INR), and thromboplastin test were done. Radiological investigations like chest X-ray and ultrasonography of the abdomen were also done. The clinical course of each patient was followed, and outcomes were documented.

Statistical analysis

Statistical analysis was performed using the latest version of IBM SPSS Statistics software (version 29.0; IBM Corp., Armonk, NY). Descriptive statistics, including proportions and percentages, were used to summarize categorical variables such as demographic characteristics and clinical features. The chi-square test was applied to assess associations between categorical variables, such as the relationship between clinical symptoms and disease severity. To identify independent predictors of adverse outcomes, multivariate logistic regression analysis was employed.

## Results

Participants (*n* = 105) were classified based on disease severity into two groups: non-severe dengue (*n* = 90, 85.7%) and severe dengue (*n* = 5, 14.3%). The mean age of patients was 92.02 months, with the majority (*n* = 48, 45.7%) aged 60-120 months. Males comprised 68.6% (*n* = 72) of the cohort. Both age and sex distributions did not show significant associations with disease severity. Most patients were admitted between three and six days of illness (*n *= 48, 45.7%), with a mean admission day of 5.08. The average hospital stay was 4.63 days, with severe cases requiring prolonged admission (more than three days in 93.3% of severe cases). Out of the total patients enrolled, 101 (96.3%) were successfully discharged following recovery. Among these, 90 had non-severe dengue, while 11 with severe dengue also recovered, indicating favorable outcomes even in some severe cases with appropriate treatment. One patient (0.9%) was referred to a higher healthcare facility, and three patients (2.8%) succumbed to the illness, all of whom had severe dengue (Table 1).

**Table 1:** Demographic profile.

Parameter	Variables	No.	%	Non-severe dengue	Severe dengue	Mean
Age (months)	≤12	10	9.5	7	3	92.02
	12-60	15	14.3	14	1	
	60-120	48	45.7	43	5	
	>120	32	30.5	26	6	
Sex	Male	72	68.6	62	10	-
	Female	33	31.4	28	5	
Duration of stay (days)	0-3	18	17.1	17	1	4.63
	3-6	60	57.1	51	9	
	>6	27	25.7	22	5	
Days of admission (days)	0-3	35	33.3	31	4	5.08
	3-6	48	45.7	40	8	
	>6	22	21	19	3	
Outcome	Discharge	101	96.3%	90	11	
	Referred	01	0.9%	00	01	
	Death	03	2.8%	00	03	
Classification	Dengue fever without warning signs	68	64.8			
	Dengue fever with warning signs	22	21			
	Severe dengue	15	14.3			

Of the total 105 patients, 68 (64.8%) had dengue without warning signs, 22 (21.0%) had dengue with warning signs, and 15 (14.3%) had severe dengue. All three patients who died from dengue-related complications had a duration of illness of five days at the time of admission. All patients presented with refractory shock, indicating severe circulatory compromise. Two had significant bleeding manifestations; one had severe thrombocytopenia, and both showed elevated INR, indicating coagulopathy. One patient had developed acute kidney injury (AKI) with elevated creatinine levels, and another had signs of altered sensorium, suggesting possible central nervous system involvement. Additionally, one patient had pleural effusion with radiological evidence of pneumonia on chest X-ray. These findings shows that the presence of multiple organ dysfunctions and severe complications at presentation, contributing to the fatal outcome.

Leukocyte counts, platelet levels, and hematocrit did not differ significantly between severity groups. Elevated SGPT levels (>50 IU/L) were significantly more common in severe cases (7, 46.67%, vs. 21, 23.33%; *P* = 0.002). SGOT elevation was also more frequent in severe dengue (9, 60.00%, vs. 35, 38.89%), though not statistically significant (*P* = 0.058). Chest X-ray abnormalities were noted to be significantly associated with severe dengue (6, 40.00%, vs. 7, 7.78%; *P* = 0.000), whereas abdominal ultrasound findings, including hepatomegaly, ascites, and gallbladder wall edema, were not significantly different between groups, although normal ultrasound findings were less common in severe dengue (13.33% vs. 36.67%; *P* = 0.139). A positive tourniquet test was found to be significantly more frequent in severe dengue (9, 60%, vs. 9, 10%; *P* < 0.000) (Table 2).

**Table 2:** Correlation of severity of disease with investigations. *P*-value < 0.05 was significant. TLC, total leucocyte count; SGPT, serum glutamic pyruvic transaminase; SGOT, serum glutamic oxaloacetic transaminase; USG, ultrasonography

Investigation	Variation	Non-severe dengue (*n *= 90)	Severe dengue (*n *= 15)	Total (*n *= 105)	*P*-value
TLC (cells/mm^3^)	Leukopenia (<4,000 cells/mm^3^)	18 (20.0%)	2 (13.33%)	20 (19.05%)	0.304
	Normal (4,000-11,000 cells/mm^3^)	57 (63.33%)	8 (53.33%)	65 (61.90%)	
	Leukocytosis (>11000 cells/mm^3^)	15 (16.67%)	5 (33.33%)	20 (19.05%)	
Raised SGPT (>50 IU/L)	Total	21 (23.33%)	7 (46.67%)	28 (26.67%)	0.002
	50-200 U	19 (90.48%)	2 (85.71%)	21 (89.29%)	
	200-1,000 U	0 (0%)	2 (14.29%)	2 (3.57%)	
	>1,000 U	2 (9.52%)	3 (0%)	5 (10.71%)	
Raised SGOT (>50 IU/L)	Total	35 (38.89%)	9 (60.00%)	44 (41.90%)	0.058
	50-200 U	30 (85.71%)	5 (88.89%)	35 (86.36%)	
	200-1,000 U	3 (8.57%)	1 (11.11%)	4 (9.09%)	
	>1,000 U	2 (5.71%)	3 (0%)	5 (4.55%)	
Platelet count	>150,000	22 (24.44%)	2 (44.44%)	24 (24.76%)	0.236
	150,000-100,000	23 (25.56%)	4 (55.56%)	27 (26.67%)	
	100,000-50,000	33 (36.67%)	4 (55.56%)	37 (36.19%)	
	<50,000	12 (13.33%)	5 (11.11%)	17 (12.38%)	
Hematocrit	≥36.3%	37 (41.11%)	6 (40%)	43 (40.95%)	0.930
	<36.3%	53 (58.89%)	9 (60%)	62 (59.05%)	
Chest X-ray	Abnormal (B/L pleural effusion, right-sided effusion, left-sided effusion, lung consolidation)	07 (7.78%)	06 (40%)	13 (12.3)	0.000
	Normal	83 (92.22%)	09 (86.67%)	92 (87.6%)	
USG abdomen	Hepatomegaly	21 (23.33%)	05 (33.33%)	26 (24.76%)	0.612
	Ascites	23 (25.55%)	05 (33.33)	28 (24.76%)	0.753
	Gallbladder wall edema	13 (14.44%)	03 (20%)	16 (14.29%)	0.868
	Normal	33 (36.67%)	02 (13.3%)	35 (36.19%)	0.139
Torniquet test	Positive	09 (10%)	09 (60%)	18 (17.14%)	<0.000
	Negative	81 (90%)	06 (40%)	87 (82.86%)	
Dengue serology	NS1 positive	54	11	65	0.327
	IgM positive	47	09	56	0.578
	Both NS1 and IgM positive	11	05	16	0.036

Dengue serology (NS1 and IgM) patterns were similar across severity groups, except for cases positive for both NS1 and IgM, which were more frequent in severe dengue (*P* = 0.036) (Table 2).

Bleeding was observed in 10 (9.5%) patients and was more prevalent in severe dengue. All bleeding cases had an elevated INR (>1), while none of the patients with normal INR showed bleeding (*P* = 0.057). Thrombocytopenia and elevated liver enzymes were common among bleeding patients but were not statistically significant (Table 3).

**Table 3:** Correlation of bleeding manifestation with investigations. *P*-value < 0.05 was significant. INR, international normalized ratio; *n*, number of subjects

		Bleeding manifestations			*P*-value
		Present (*n* = 10)	Absent (*n *= 95)	Total (*n *= 105)	
Thrombocytopenia (<1.5 lac/mm^3^)	Present	07	72	79	0.688
	Absent	03	23	26	
Hepatomegaly	Present	03	23	26	0.688
	Absent	07	72	79	
Raised SGOT (>50 IU/L)	Present	05	39	44	0.587
	Absent	05	56	61	
Raised SGPT (>50 IU/L)	Present	03	25	28	0.473
	Absent	05	72	77	
Raised INR (>1)	Present	10	69	79	0.057
	Absent	00	26	26	

**Figure 1:**
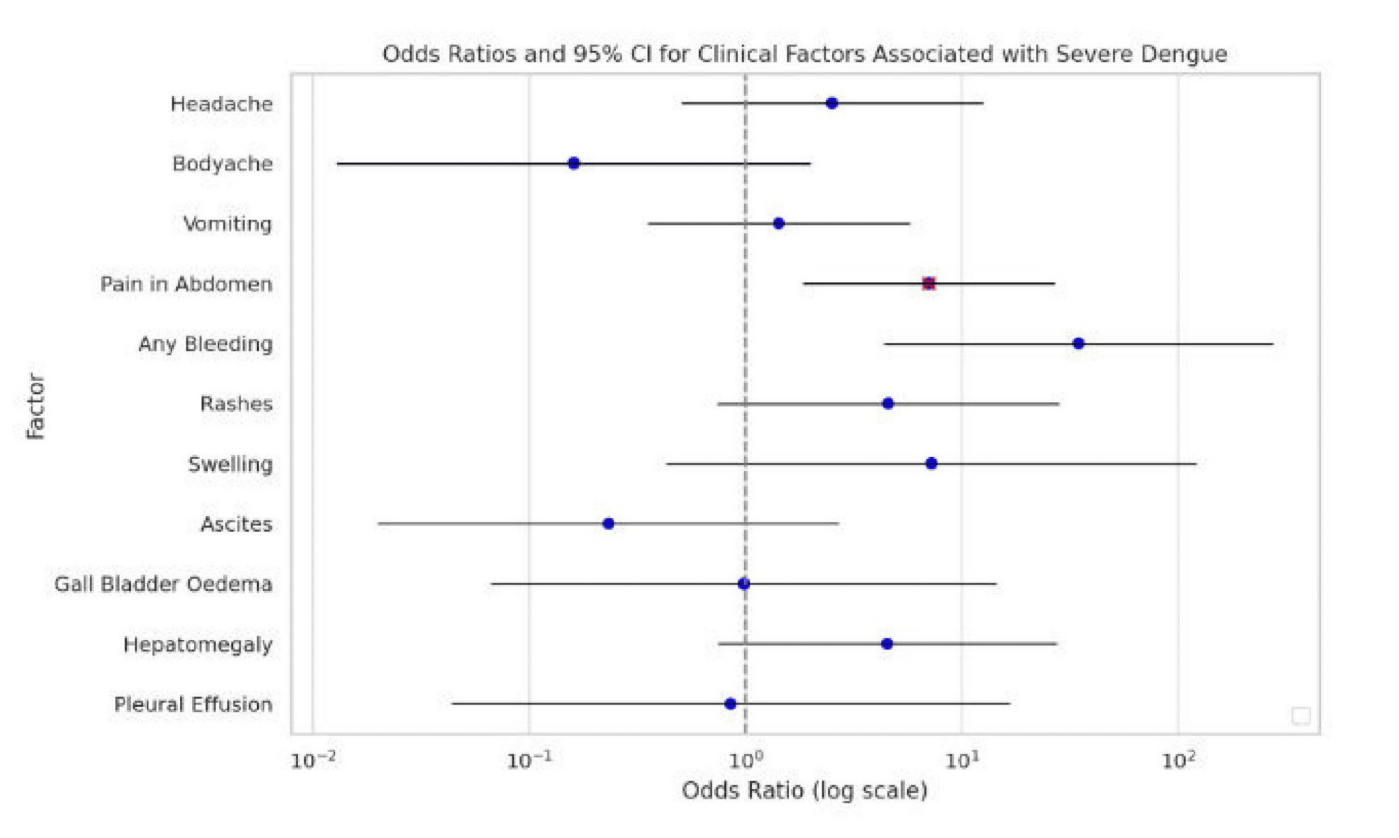
Forest plot representing the odds ratios (OR) and 95% confidence intervals (CI) for clinical factors associated with severe dengue.

On univariate logistic regression, bleeding manifestations had the highest odds ratio (OR) for severe dengue (OR = 34.783; 95% confidence interval [CI]: 4.399-275.053; *P* = 0.101), though not statistically significant. Other clinical features such as rash (OR = 4.574), swelling (OR = 7.254), and hepatomegaly (OR = 4.531) also showed elevated odds without statistical significance. Abdominal pain showed a statistically significant positive association with severe dengue (OR = 7.059; 95% CI: 1.861-26.777); *P* = 0.004) in both univariate and multivariate analyses (Tables 4-5; Figure 1).

**Table 4:** Clinical features.

Symptoms	Number of patients	%
Fever	105	100
Headache	22	20.95
Body ache	30	28.57
Vomiting	47	44.76
Pain in the abdomen	46	43.81
Any bleeding	10	9.52
Rashes	14	13.33
Swelling	4	3.81
Altered sensorium	1	0.95
Seizures	1	0.95

**Table 5:** Logistic regression analysis of risk factors for severe dengue. *P*-value < 0.05 was significant. CI, confidence interval

Factors	Odds ratio	95% CI		*P*-value
		Lower	Upper	0.258
Headache	2.516	0.509	12.438	0.154
Body ache	0.161	0.013	1.986	0.616
Vomiting	1.426	0.356	5.722	0.281
Pain in the abdomen	7.059	1.861	26.777	0.004
Any bleeding	34.783	4.399	275.053	0.101
Rashes	4.574	0.744	28.115	0.168
Swelling	7.254	0.433	121.399	0.242
Ascites	0.233	0.020	2.681	0.991
Gallbladder edema	0.984	0.067	14.440	0.100
Hepatomegaly	4.531	0.748	27.464	0.917
Pleural effusion	0.854	0.044	16.600	0.258

## Discussion

In this study, out of the 105 laboratory-confirmed dengue cases, 90 (85.7%) were classified as non-severe dengue and 15 (14.3%) as severe dengue, according to WHO classification [8]. The mean age of patients was 92.02 months, with nearly half (48, 45.7%) aged between 60 and 120 months, and a male predominance (72, 68.6%), findings consistent with studies from Southern India and Odisha reporting similar age distributions and male predominance, but in contrary to study by Adnan et al. who found a female preponderance [8-10]. Most patients presented between days 3 and 6 of illness, aligning with prior reports of early hospital presentation [11]. The average hospital stay was 4.63 days, with prolonged admissions seen in 93.3% of severe cases, which was comparable to a study by Alam et al. [12]. Clinical recovery was achieved in 101 cases (96.3%), comparable to the 97.5% recovery rate reported in other pediatric studies [8]. Mortality in our cohort was 3 cases (2.8%), observed exclusively among patients with severe dengue, and was slightly lower than that reported in studies from Bangladesh and North India [11-12]. These findings show the generally favorable outcomes in pediatric dengue with timely intervention, even in severe cases. A possible shift in the seasonal distribution of dengue was observed, with many cases occurring in the winter season.

Fever was reported in all patients (105, 100%), consistent with observations across multiple studies where fever was the predominant presenting symptom in pediatric dengue cases [8,12]. Gastrointestinal symptoms such as vomiting (47, 44.76%) and abdominal pain (46, 43.81%) were also highly prevalent in our study, aligning with findings from Southern Odisha and North India, where such symptoms were reported in 40%-60% of children [9,11].

Skin manifestations, including rash, were present in 14 patients (13.33%), which is lower than the 20-35% range reported in other studies [9,13]. Bleeding manifestations were observed in 10 patients (9.52%), a proportion comparable to that reported by Daniel et al. in Kerala and by Sahana and Sujatha in Southern India, indicating a consistent trend of low to moderate hemorrhagic presentation among children [8,13]. Neurological features such as altered sensorium and seizures were rare (0.95% each). Overall, our findings were in concordance with the existing literature, reinforcing the predominance of fever and gastrointestinal symptoms in pediatric dengue, with variable but generally low incidence of bleeding and neurological involvement.

Significantly elevated levels of SGPT were observed in severe dengue cases (7, 46.67%) compared to non-severe cases (21, 23.33%; *P* = 0.002), supporting prior reports that liver dysfunction is a prominent feature of severe infection. Elevated SGOT levels were also more frequent in severe cases in our cohort (9, 60.0%, vs. 35, 38.89%), although this was not statistically significant (*P* = 0.058). Similar results were found by other studies where patients had raised liver enzymes [8,14]. These findings reinforce hepatic involvement as a key marker of disease severity.

Thrombocytopenia (<1.5 lakh/mm³) was documented in 72 patients (75.2%), indicating a high prevalence. However, unlike previous studies that have associated thrombocytopenia with severe dengue, we did not observe a statistically significant difference in platelet counts between the two groups (*P* = 0.236) [9,11,13]. Bleeding manifestations were seen in only seven patients (8.9%) with thrombocytopenia. Interestingly, 11.5% of patients without thrombocytopenia also exhibited bleeding, highlighting that bleeding risk in dengue may not be directly correlated with low platelet count alone. The difference may stem from differences in the timing of sample collection, local epidemiological patterns, or the small number of severe cases in our cohort.

Various radiological abnormalities were observed, like pleural effusion or consolidation on chest X-ray, and hepatomegaly, ascites, and gallbladder edema on abdominal ultrasound. Chest X-ray findings (pleural effusion or consolidation) were significantly elevated in the severe dengue group (6, 40.0%) compared to the non-severe group (7, 7.78%; *P* = 0.000), corroborating previous reports of plasma leakage and pulmonary involvement in severe dengue [9]. However, abdominal ultrasound findings such as hepatomegaly, ascites, and gallbladder wall edema were not significantly different, in contrast to findings of the study by Sahana and Sujatha [8]. It may be because of the reason that abdominal imaging may have limited discriminative value unless performed at specific phases of illness.

A notable observation in our study was the significant association of a positive tourniquet test with severe dengue (9, 60.0%) compared to non-severe cases (6, 10.0%; *P* < 0.000). While not consistently emphasized across studies, the presence of a positive tourniquet test was also seen in a tertiary care study from Northern India in adults, suggesting its potential role as a simple clinical marker of severity due to capillary fragility [15].

Among patients with bleeding manifestations (10, 9.5% of the total), elevated INR (greater than 1) was universally present, though not statistically significant (*P* = 0.057), suggesting a trend toward coagulopathy. Our findings mirror those of other studies, which have documented bleeding and hepatic involvement as key features of severe disease [9,16].

In logistic regression analysis, most clinical variables, including rash, hepatomegaly, and swelling, showed increased odds of association with severe dengue but did not achieve statistical significance. Interestingly, abdominal pain showed a significant positive association with severe dengue (*P* = 0.004). This is consistence with earlier studies, which typically reported abdominal pain as more common in severe cases [8,9].

Dengue serology patterns, including NS1 and IgM positivity, did not significantly differ between groups, but both NS1 and IgM positivity were seen more commonly in severe cases (*P* = 0.036). Although serological trends are inconsistently reported across studies, they may reflect variations in the immune response and timing of testing.

This study has certain limitations. Only hospitalized dengue cases were included in the study. The study duration was six months only; extending the study to a whole year would provide more details about the occurrence of dengue in different months around the year. The age group of the study was 0-14 years, so the study provides only limited data on adolescent children of dengue. The study could not provide details of the serotypes of the dengue virus. Those children who died before testing could not be enrolled in the study, which led to survival bias. Though these limitations may impact the generalizability of the study, it provides valuable insight into the changing clinical pattern of dengue in children in India.

## Conclusions

This study supports that the majority of pediatric dengue cases fall into the non-severe category, with fever and gastrointestinal symptoms being the most typical presentations. Although thrombocytopenia was a common finding, it did not emerge as a reliable indicator of disease severity. On the other hand, severe dengue was found to be more closely linked to raised liver enzymes, a positive tourniquet test, and radiological abnormalities. These results highlight the importance of a comprehensive clinical approach that takes into account parameters other than hematocrit and platelet counts to identify these cases early. A high index of suspicion is needed for cases presenting with atypical features or occurring outside the usual seasonal patterns.
